# Survivin Overexpression Is Associated with Aggressive Clinicopathological Features in Cervical Carcinoma: A Meta-Analysis

**DOI:** 10.1371/journal.pone.0165117

**Published:** 2016-10-20

**Authors:** Ke-yan Cheng, Zhi-lian Wang, Qian-yun Gu, Min Hao

**Affiliations:** Department of Obstetrics and Gynecology, The Second Hospital of Shanxi Medical University, Taiyuan, Shanxi Province, China; Heinrich-Heine-Universitat Dusseldorf, GERMANY

## Abstract

**Objective:**

Overexpression of survivin has been reported in many human tumors. However, the clinicopathological features associated with survivin overexpression in cervical carcinoma remain controversial. Thus, the current meta-analysis was performed to assess the clinicopathological significance of survivin in cervical carcinoma.

**Methods:**

PubMed, EMBASE, and Web of Science databases were searched for relevant studies published through November 1, 2015. A meta-analysis was performed to evaluate the association between survivin expression and clinicopathological outcome in cervical carcinoma.

**Results:**

Eleven eligible studies with a total of 865 patients were included. Survivin overexpression was closely related to lymph node metastasis (odds ratio [OR] = 0.679, 95% confidence interval [CI]: 0.509–0.905, P = 0.008) but was not significantly associated with tumor FIGO stage (I+II vs. III+IV) (OR = 0.843, 95% CI: 0.626–1.137, P = 0.264), tumor grade (G1+G2 vs. G3) (OR = 0.913, 95% CI: 0.689–1.210, P = 0.527), tumor size (>4 vs. ≤4 cm) (OR = 0.825, 95% CI: 0.434–1.570, P = 0.559), or stromal involvement (OR = 0.820, 95% CI: 0.545–1.233, P = 0.340). The correlation between survivin expression and overall survival was evaluated among a total of 238 patients from three eligible studies. The pooled HR was 1.129 (95% CI: 0.597–1.661; P = 0.000), indicating that survivin expression was significantly associated with poor survival in cervical carcinoma.

**Conclusions:**

Based on the current meta-analysis, survivin is strongly associated with lymph node metastasis and poor prognosis. Additionally, survivin is a novel clinicopathological marker of cervical carcinoma and thus may be a therapeutic target for cervical carcinoma.

## Introduction

Cervical carcinoma is the main lethal malignancy affecting the female reproductive system. An estimated 70,000 deaths result from cervical carcinoma annually, and 500,000 cases are newly diagnosed each year [1]. Although therapeutic options have improved, the treatment of lymph node metastasis and locally advanced tumors remains a major challenge [2]. Moreover, recurrent disease develops in more than 70% of patients with lymph node metastasis but only approximately 10–20% of patients without advanced cervical carcinoma [3]. Therefore, identifying clinicopathological markers to predict lymph node metastasis and the development of malignant tumors may reveal potential therapeutic targets for cervical carcinoma.

One protein of increasing interest is survivin, a unique member of the inhibitor of apoptosis (IAP) protein family. Survivin is expressed in many malignant tumors, including those affecting lung, liver, breast and the gastrointestinal system [4–7], but is undetectable in nonproliferating adult tissues [8]. Survivin is considered a novel clinicopathological marker for numerous human malignant tumors. Moreover, survivin expression has been correlated with clinical outcome. During the G2/M phase of the cell cycle, survivin plays a crucial role in regulating cell division, overcoming the apoptotic checkpoint, and inhibiting caspase-3 and caspase-7 activity [9–12]. Therefore, survivin inhibits apoptosis, enhances proliferation, and promotes angiogenesis [9,11,12].

However, the clinicopathological features associated with survivin expression in cervical carcinoma remain controversial. To more precisely evaluate the relationship between survivin expression and clinicopathological outcome in cervical carcinoma, we conducted a meta-analysis of 11 published studies.

## Methods

### Literature search

The PubMed, EMBASE, and Web of Science databases were systematically searched for relevant studies until November 1, 2015. The following key words were used individually and in combination: “cervical cancer”, “cervical carcinoma,” “cancer of cervix”, and “survivin”. The reference lists of the selected articles were reviewed to identify additional studies not found in the original search.

### Inclusion and exclusion criteria

Two researchers reviewed the generated list of unique articles for studies that met the following inclusion criteria: 1) a study focus on the association between survivin and clinicopathological variables; (2) immunohistochemistry (IHC) or real-time PCR (RT-PCR) analysis to evaluate survivin expression in cervical carcinoma; (3) the use of odds ratios (ORs) with 95% confidence intervals (CIs) to analyze provided or extracted clinicopathological variables; (4) comparison of overall survival between different levels of expression of survivin in cervical cancer; (5) hazard ratio (HR) and 95% CI for overall survival according to survivin status were reported or could be computed from the data presented. In cases of overlapping patient cohorts presented over multiple studies, the study with the largest or most recent dataset was employed. The following studies were excluded: (1) letters, reviews, case reports, conference abstracts, or expert opinions; and (2) articles with insufficient information on clinicopathological variables.

### Data extraction

Two investigators independently extracted data that met our inclusion and exclusion criteria. Any discrepancies were resolved by consensus. The following information was extracted: first author, publication year, country, detection method, cutoff value, tumor size, numbers of cases and controls, clinicopathological variables, antibody source, HR estimate.

### Assessment of study quality

The Newcastle-Ottawa Scale (NOS) was used to evaluate the methodological quality of all included cohort studies [13]. Three grouping categories were used with the NOS: selection, comparability and exposure/outcome measurement. A study was awarded a maximum of one star for each numbered item within the selection and outcome categories and a maximum of two stars for comparability. The total number of stars was counted for each study, and studies with more stars were of higher methodological quality. A study could receive a maximum of nine stars.

### Statistical analysis

We extracted and summarized data on survivin expression associated with cervical carcinoma from the included studies. Pooled estimates of ORs with 95% CIs were used to evaluate the associations between survivin expression and clinicopathological features of cervical cancer. To stratify the data for analysis, the survivin expression and clinicopathological parameter results were combined into single categories with comparable clinicopathological relevance. These categories included presence of lymph node metastasis, tumor FIGO stage (I+II vs. III+IV), tumor grade (G1+G2 vs. G3), tumor size (>4 vs. <4 cm), and presence of stromal involvement. The HR and 95% CI were used to estimate the impact of survivin expression on overall survival. HR and its variance for each individual study were extracted or calculated based on the published studies according to the methods described by Parmar [14]. Kaplan-Meier curves were read by Engauge Digitizer version 4.1 (http://digitizer.sourceforge.net/). Based on the chi-squared statistic Q, inter-study heterogeneity was assumed [15] in cases in which I^2^>50%, and ORs were pooled according to random-effects models. Alternatively, fixed-effects models were used. We conducted a sensitivity analysis to identify the summary effect estimate. In addition, Begg’s funnel plots and Egger’s test were used to statistically assess publication bias [16]. All statistical analyses were performed using Stata 13.0 (Stata Corporation, College Station, TX). A p-value less than 0.05 was considered statistically significant.

## Results

### Study selection and characteristics

**Fig 1:**
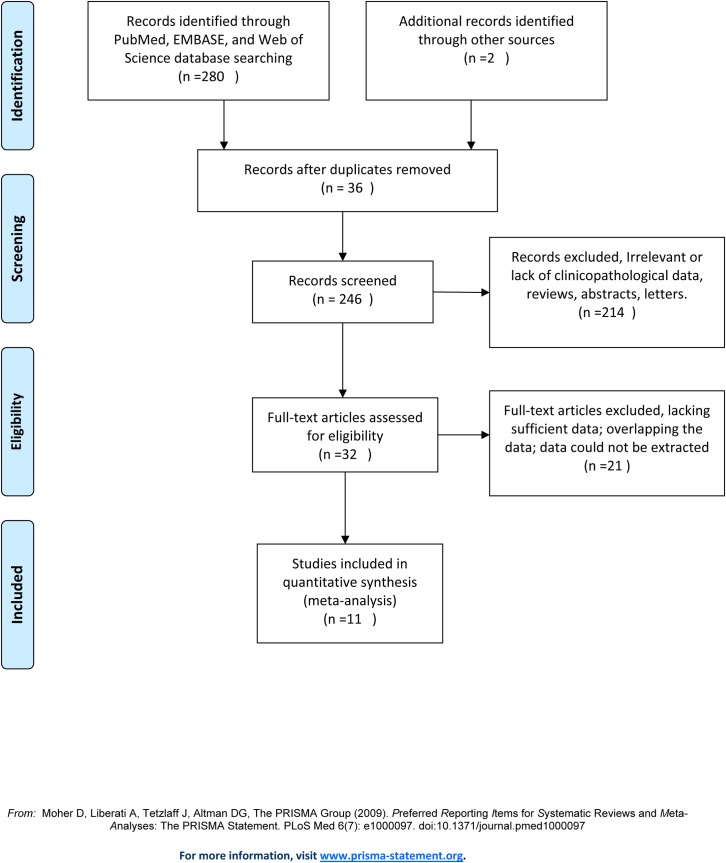
Flow chart of study selection process for the meta-analysis.

A total of 282 potentially relevant studies were retrieved after the initial database search. Following title and abstract screening, 36 records were excluded due to duplication. Another 214 studies were excluded due to a lack of focus on cervical carcinoma, an absence of clinicopathological data, or not survival. After reading 32 full-text articles, 21 additional studies were excluded: 12 studies lacked sufficient data, 2 studies presented overlapping data, and 7 studies had data that could not be extracted. Overall, 11 studies were finally included in the meta-analysis (Fig 1).

The 11 included studies comprised a total of 865 patients. The majority of the cohorts were in Asia. The number of patients per study ranged from 41 to 142. The percentage of positive survivin expression ranged from 67% to 96%. Most of the included studies focused on stage I–IV cancer (n = 7). Nine studies utilized IHC, and 2 studies utilized RT-PCR. Survivin expression was evaluated in two tissue groups: squamous cell carcinoma (SCC) (n = 5) and SCC plus adenocarcinoma (ADC) (n = 6). The detected survivin expression was primarily localized to the cytoplasm of tumor cells (n = 8). In 3 studies, nuclear-localized expression was observed. The main characteristics of the included studies are presented in Table 1.

**Table 1:** Characteristics and results of the included studies.

Study	Year	Country	No. of P.	No. of positive (%)	Method	Location	Cutoff V.	Sample	Stage	Clinicopathological variables	NOS	Antibody source	HR Estimate
Lee J.P[17]	2005	Korea	64	51/53(96%)	IHC	Nu.& Cyt.	20%	SCC	I-IV	D,LN,T,M,S	8	R&D	Sur. Curve
H. Zhu [18]	2010	China	101	63/81(78%)	RT-PCR	Cyt.	5%	SCC&ADC	I-III	D,LN,M,T,S	7	Boster	Sur. Curve
H. Lu [19]	2010	China	142	90/107(84%)	IHC	Cyt.	5%	SCC&ADC	I-IV	D,LN,T,S	8	Gene Company Ltd	Sur. Curve
X.Q. Cao [20]	2014	China	116	72/81(88%)	RT-PCR	Cyt.	10%	SCC&ADC	I-IV	D,LN,T,S	8	Novus Biologicals	NA
H.Q. Liu [21]	2015	China	80	40/50(80%)	IHC	Nu.& Cyt.	5%	SCC	I-II	D,LN,T,S	8	Labvision	Sur. Curve
S. Lu [22]	2005	China	51	32/41(78%)	IHC	Cyt.	5%	SCC&ADC	I-IV	D,LN,M,S	6	Jinmen	NA
M. Wang [23]	2001	China	69	41/59(69%)	IHC	Cyt.	10%	SCC	I-III	D,S	6	Santa Cruz	NA
Y.Q. Mu [24]	2007	China	75	45/50(90%)	IHC	Cyt.	10%	SCC&ADC	I-IV	D,LN,S	6	Maixin Biologic	NA
D. Lu [25]	2012	China	59	35/49(71%)	IHC	Cyt.	10%	SCC	I-IV	D,LN,S	7	Santa Cruz	NA
Y. Lan [26]	2005	China	41	26/31(84%)	IHC	Nu.& Cyt.	10%	SCC&ADC	I-IV	D,S	6	Neomaker	NA
S.F. Wu [27]	2012	China	67	32/47(67%)	IHC	Cyt.	10%	SCC	I-III	D,LN,S	7	Santa Cruz	NA

No. of P, number of patients; NOS, Newcastle-Ottawa quality assessment scale; RT-PCR, reverse transcription polymerase chain reaction; IHC, immunohistochemistry; Nu, nucleus; Cyt, cytoplasm; NA, not applicable; SCC, squamous cell carcinoma; ADC, adenocarcinoma; D, histological differentiation; LN, lymph node metastasis; T, depth of tumor invasion; M, metastasis; S, stage; Sur. Curve, survival curve.

### Heterogeneity assessment and meta-analysis

**Fig 2:**
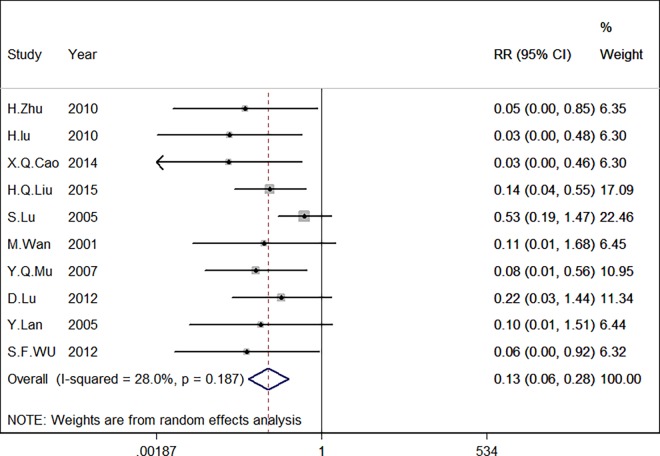
Forest plot of the pooled risk ratio (RR) for survivin expression in cervical carcinoma. Highly significant heterogeneity was observed prior to the exclusion of Lee’s study.

Highly significant heterogeneity was detected when all eligible studies were pooled (chi-squared = 35.73, I^2^ = 72.0%, p<0.001). Based on the level of heterogeneity, a random-effects model was employed for statistical analysis. We also performed sensitivity analysis to explore the source of heterogeneity. We determined that one study, an investigation conducted by Lee [17], was the main source of heterogeneity. After removing this study from our meta-analysis, the heterogeneity dropped from 72.0% to 28.0% with no significant changes in the combined effect and little change in the P value. This analysis confirmed the stability of our results, as shown in Table 2. After excluding the indicated study by Lee J.P, a meta-analysis was performed on the remaining 10 studies. The pooled RR for the eligible studies was 0.132 (95% CI: 0.062–0.282, I^2^ = 28% P<0.001). This finding suggests that survivin expression is significantly associated with cervical carcinoma (Fig 2).

**Table 2:** Sensitivity analysis.

Sensitivity analysis	Heterogeneity		Combined effect	
	I^2^	tau^2^	OR and 95% CI	P
Including Lee J.P. study	72%	1.6959	0.144 (0.054–0.387)	0
Excluding Lee J.P. study	28%	0.3911	0.132 (0.062–0.282)	0

### Correlating survivin expression with clinicopathological parameters

**Fig 3:**
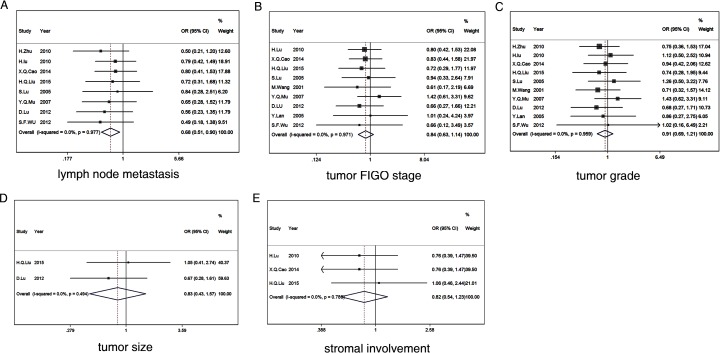
Forest plot depiction of survivin expression and odds ratios (ORs) for the following clinical pathological features: lymph node metastasis (A), tumor FIGO stage (B), tumor grade (C), tumor size (D), stromal involvement (E).

We examined the relationship between high levels of survivin expression and clinicopathological features in patients with cervical carcinoma. Survivin expression was significantly correlated with lymph node metastasis (OR = 0.679, 95% CI: 0.509–0.905, P = 0.008) (Fig 3A). However, survivin overexpression was not significantly associated with tumor FIGO stage (I+II vs. III+IV) (OR = 0.843, 95% CI: 0.626–1.137, P = 0.264) (Fig 3B), tumor grade (G1+G2 vs. G3) (OR = 0.913, 95% CI: 0.689–1.210, P = 0.527) (Fig 3C), tumor size (>4 cm vs.<4 cm) (OR = 0.825, 95% CI: 0.434–1.570, P = 0.559) (Fig 3D), or stromal invasion (OR = 0.820, 95% CI: 0.545–1.233, P = 0.340) (Fig 3E). The associations between survivin expression and several clinicopathological parameters are shown in Fig 3.

An appropriate statistical model was selected to evaluate the combined data. We evaluated all clinicopathological variables for heterogeneity, including models of lymphatic metastasis, clinical stage, stromal involvement, tumor grade, and tumor size. The results are shown in Table 3. The I^2^ values were very low, indicating a lack of significant inter-study heterogeneity. Furthermore, a significant P value (P = 0.008) was obtained only for lymph node status. This result suggests that survivin expression is significantly correlated with lymph node metastasis. No significant associations were observed between survivin expression and tumor grade (0.527), tumor FIGO stage (P = 0.264), tumor size (P = 0.559), or stromal involvement (P = 0.340).

**Table 3:** Meta-analysis evaluating the associations between survivin expression and clinicopathological variables.

Clinicopathological variable	No. of	Cases	Pooled data (fixed)			Heterogeneity test		
	studies		OR	95% CI	P value	Chi^2^	P value	I^2^(%)
Lymph node status	8	505	0.679	0.509–0.905	0.008	1.63	0.977	0.00%
FIGO stage	9	533	0.843	0.626–1.137	0.264	2.28	0.971	0.00%
Tumor grade	10	568	0.913	0.689–1.210	0.527	3.13	0.959	0.00%
Tumor size	2	99	0.825	0.434–1.570	0.559	0.47	0.494	0.00%
Stromal involvement	3	264	0.82	0.545–1.233	0.34	0.49	0.783	0.00%

### Impact of survivin expression on overall survival of cervical carcinoma patients

**Fig 4:**
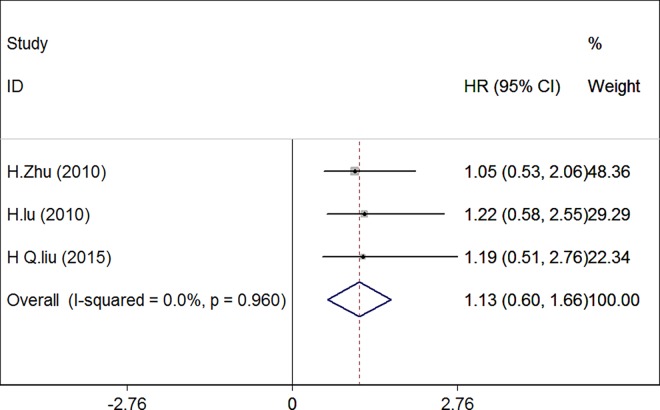
Forest plot of the summarized hazard ratios (HRs) for the association between survivin expression and overall survival in patients with cervical carcinoma.

Next, we evaluated the correlation between survivin expression and overall survival in a total of 238 patients from three eligible studies [18,19,21]. There was no significant heterogeneity among the three studies (chi-squared = 0.08, I^2^ = 0%, P = 0.960), and thus a fixed-effects model was used in the meta-analysis. The pooled HR was 1.129 (95% CI: 0.597–1.661; P = 0.000), indicating that survivin expression was significantly associated with poor survival in cervical carcinoma compared to cancer tissues exhibiting no expression of survivin. (Fig 4).

To further investigate the relationship between survivin expression and overall survival, we performed one-way sensitivity analysis. The results indicated that none of the included studies had an influence on our pooled HR or induced heterogeneity (chi-squared = 0.08, I^2^ = 0.0%, P = 0.960). Moreover, we performed subgroup analyses according to the technique used to detect survivin or the statistical method used to estimate the HR (Table 4). Both the combined HRs of studies detecting survivin by PCR (HR 1.050, 95% CI 0.285–1.815, P = 0.001) or by IHC (HR 1.203; 95% CI 0.463–1.943, P = 0.007) and the summary HRs estimate using HR (HR 1.203; 95% CI 0.463–1.943, 0.001) versus Sur. Curve (HR 1.050; 95% CI 0.285–1.815, P = 0.007) supported the observation that survivin was significantly correlated with prognosis in patients with cervical carcinoma. When stratified according to histological type, the combined HRs of both SCC and SCC&ADC showed HR = 1.186 (95% CI: 0.060–2.312, P = 0.039) and 1.113 (95% CI: 0.509–1.716, P = 0.000), separately. We also observed statistically significant effects of survivin expression on tumor stage with HRs of 1.186 (95% CI: 0.060–2.312, P = 0.039), 1.050 (95% CI 0.285–1.815, P = 0.007), and 1.216 (95% CI: 0.233–2.199, P = 0.015) for I–II, I–III, and I–IV, respectively. When we aggregated the studies according to survivin cutoff value, the pooled HR for the 5% cutoff value was 1.129 (95% CI: 0.597–1.661, P = 0.000). These results indicated that the expression of survivin remained associated with the survival of patients with cervical carcinoma.

**Table 4:** Subgroup analysis of summarized hazard ratios reflecting the relationship between survivin and overall survival in cervical cancer.

Subgroup analysis	No. of	Number of	HR (95%Cls)	P-value
	studies	patients		
Overall	3	238	1.129 (0.597–1.661)	0
HR estimate				
HR	2	157	1.203 (0.463–1.943)	0.001
Sur. curve	1	81	1.050 (0.285–1.815)	0.007
Histological type				
SCC	1	50	1.186 (0.060–2.312)	0.039
ADC & SCC	2	188	1.113 (0.509–1.716)	0
Method				
IHC	2	188	1.203 (0.463–1.943)	0.007
RT-PCR	1	81	1.050 (0.285–1.815)	0.001
Tumor stage				
I-II	1	50	1.186 (0.060–2.312)	0.039
I-III	1	81	1.050 (0.285–1.815)	0.007
I-IV	1	107	1.216 (0.233–2.199)	0.015
Cutoff value				
5%	3	238	1.129 (0.597–1.661)	0

### Sensitivity analysis

**Fig 5:**
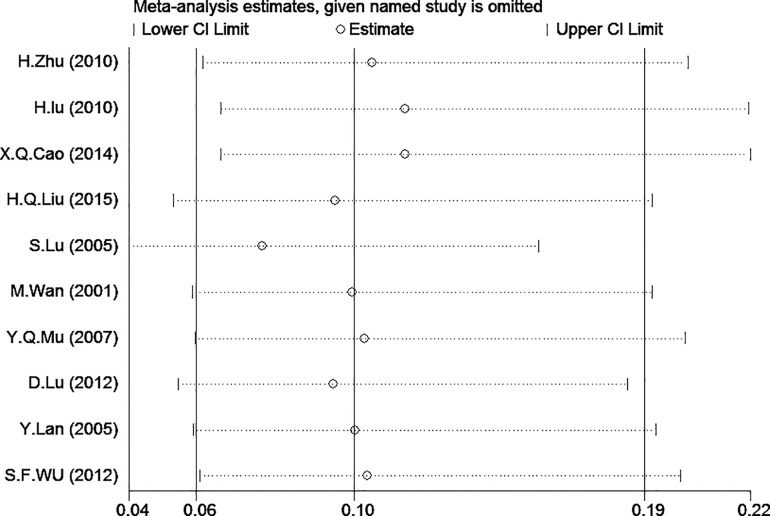
Sensitivity analysis of the summary odds ratio (OR) coefficients of the relationships between survivin expression and risk of cervical carcinoma.

We conducted a sensitivity analysis to analyze the studies with lower or lowest risk in the domain of selection bias, Sensitivity analysis indicated that the study by Lee J.P.[17] should be removed, and we then conducted the model again to determine the effect on the overall estimate. We observed that I^2^ = 72.0% decreased to I^2^ = 28.0%, with little change in the combined effect (Fig 5). The pooled ORs were not greatly influenced. Thus the results were statistically stable.

### Publication bias

**Fig 6:**
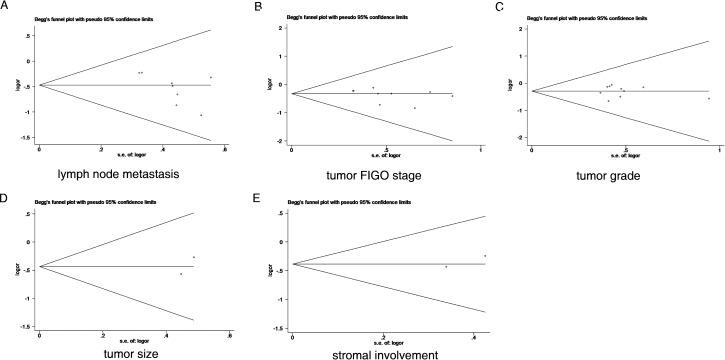
Egger’s funnel plot for the assessment of publication bias regarding the relationships between survivin expression and the following clinicopathological features: lymph node metastasis (A), tumor FIGO stage (B), tumor grade (C), tumor size (D), and stromal involvement (E).

Begg’s funnel plots and Egger’s test were used to assess publication bias in the meta-analysis. Our data produced no funnel plot asymmetry for any of the included studies (Fig 6). Egger’s test results showed no significant publication bias for lymph node status (P = 0.061), FIGO stage (P = 0.206), tumor grade (P = 0.779), tumor size (P = 0.317), or stromal involvement (P = 0.340). Furthermore, for the impact of overall survival, Egger’s test results also showed no significant publication bias (P = 0.516). Based on these results, there was no evidence of publication bias.

## Discussion

The use of survivin expression as a clinicopathological marker of malignancy has received increasing attention. Overexpression of survivin has been observed in esophageal, gastric and lung cancer tissues and represents a poor prognostic factor in these cancer patients [6,28–30]. Survivin has also been recently associated with clinical stage, cervical intraepithelial neoplasia grade and lymph node metastasis in cervical cancer [21]. Survivin is re-expressed in most cancers and is associated with tumor aggression and decreased patient survival rates [31], making it a potential target for inhibiting carcinoma progression. However, these conclusions must be confirmed in larger trials. In the current study, to evaluate the relationship between survivin expression and specific clinicopathological features of cervical carcinoma, we performed a meta-analysis of a large sample size.

Our meta-analysis included 11 studies. The pooled ORs of these studies indicated that survivin is a clinicopathological marker in cervical carcinoma. Recently, several studies have revealed that survivin plays an important role in cervical tumorigenesis. Kim et al. showed that the survivin positivity significantly increased during cervical carcinogenesis, following a gradient from low-grade squamous intra-epithelial lesions to high-grade squamous epithelial lesions and squamous tumors [32]. These studies are consistent with our observations that survivin expression is higher in cervical cancer than in normal cervical tissues.

We identified a significant association between survivin expression and lymph node metastases in patients with cervical carcinoma. Furthermore, no statistical relationship between survivin expression and other clinicopathological variables, such as stromal involvement, tumor stage, tumor size or FIGO stage, was observed. Our results are consistent with those of other studies evaluating the role of survivin as a clinicopathological marker in cervical carcinoma. For example, a report by Lu and colleagues showed that survivin overexpression is mainly related to lymph node metastasis [19]. Wu et al. reported that survivin was significantly correlated with clinical staging and lymph node metastases in cervical carcinoma [27]. This observation is best explained by the molecular mechanisms by which survivin promotes cellular proliferation, inhibits apoptosis, and increases angiogenesis [9,11,12], which have been extensively explored in cancer cells. For instance, McKenzie et al. observed that survivin overexpression enhanced migration on fibronectin and invasion through Matrigel, whereas survivin knockdown under sub-apoptotic conditions blocked migration and invasion [33]. Kogo et al. also showed that YM155, a small-molecule survivin inhibitor, significantly suppressed tumor growth and lymph node metastasis in cervical cancer cells [34].

The molecular mechanisms of survivin may also be partly attributable to the heterodimerization of survivin with its splice variants in tumor cells. The survivin gene has four dominant (1, 2, 3, and 4) and two hidden (2B and 3B) exons. Alternative splicing of its pre-mRNA produces four different mRNAs that encode four distinct proteins: survivin, survivin-2B, survivin-△Ex3, and survivin-3B [34,35]. Some of these splice variants are differentially expressed in various cancers and have been reported to correlate with clinical parameters, including outcome. In breast and thyroid cancers, survivin△Ex3 has been associated with invasion and malignancy [36,37]. In gastric cancer, survivin-2B expression was significantly decreased in later tumor stage (III+IV) compared with early stage (I+II) and inversely correlated with tumor differentiation and invasion [38]. Moreover, although Krieg et al. indicated that survivin and survivin-△Ex3 remained unchanged in different stages of cancer, Meng et al. showed that the expression level of survivin-△Ex3 was inversely correlated with the apoptotic index [39]. In addition, survivin-△Ex3 and survivin-2B may play opposing roles in tumor progression and/or tumorigenesis [40]. In our meta-analysis, the antibodies used in most studies do not discriminate between survivin isoforms, and thus the results may reflect increases in total survivin levels, including all known isoforms. Thus, more studies are needed to assess the prognostic significance of these results.

We also evaluated the correlation between survivin expression and overall survival in a total of 238 patients from three eligible studies. Survivin expression was significantly associated with poor survival in cervical carcinoma. Our analysis is supported by clinical observations that high survivin is associated with advanced disease, metastatic disease and poor prognosis in esophageal, gastric and lung carcinoma tissues [6,28–30]. Werner et al. reported that high survivin expression was associated with advanced disease and poor prognosis in medullary thyroid carcinoma [41]. Kawasaki et al. reported that survivin expression was associated with reduced apoptotic index and significantly worse survival rates in colorectal carcinomas [42]. These observations all suggest that survivin is an independent prognostic factor.

Further, when stratifying the included HR data, we also observed significant associations between detection method, tumor type, cutoff value, histological type, and tumor stage. These results indicated that survivin expression was significantly associated with poor survival in cervical carcinoma. Our data indicate that although IHC was influenced by various factors, IHC (P = 0.007) and PCR (P = 0.001) are equally important for the analysis of survivin expression. With respect to tumor type and histological type characteristics, Hong et al. reported that primary tumors of ADC displayed a slower and a poorer response than those of SCC for patients treated with primary irradiation. Patients with ADC also had lower a 5-year disease-specific survival rate than those with SCC [43]. Consistent with our data, P-values were 0.000 and 0.039 in ADC and SCC, respectively. With respect to tumor stage, Cao et al. reported that survivin overexpression participates in the occurrence and development of cervical cancer. Survivin and clinical stage were independent prognostic factors in cervical cancer [20]. In summary, survivin overexpression is clearly associated with metastatic lymph node metastasis and poor prognosis in cervical carcinoma, and survivin may represent a therapeutic target in patients with cervical carcinoma.

Based on the above analysis, we believe that our results are robust. One study [17] was excluded from our meta-analysis due to significant heterogeneity, but the exclusion of this study produced no significant changes in the combined effect or P value of the meta-analysis. Collectively, the heterogeneity analysis, sensitivity analysis and assessment of publication bias produced robust and credible results.

However, several limitations of our study must be addressed. First, the cervical carcinoma patients in the included studies were of Asian descent. Whether the results are applicable to other races is unknown, and future studies should include other races. Second, the role of survivin within a cell can be affected by its subcellular location [44]. Suzuki et al. reported that cytoplasmic survivin-negative or a combination of nuclear survivin-positive and cytoplasmic survivin-negative was correlated with a favorable prognosis for local control in cervical squamous cell carcinoma patients treated with radiation therapy alone [45]. In our meta-analysis, the majority of included studies did not indicate whether survivin was expressed in the nucleus or cytoplasm; this lack of differentiation potentially affected the interpretation of the provided data. Third, the techniques used to detect survivin may have differed between the included studies. In the meta-analysis, IHC staining was used to assess survivin expression. Although IHC staining is a simple, effective method, its results are highly influenced by a variety of factors, such as the length of sample storage, antibody, fixation method for paraffin-embedded samples, and cutoff level for a positive signal [46]. Therefore, to avoid some selection bias, we included a methodological assessment of the studies. We determined that IHC and RT-PCR are equally important for the detection of survivin. Fourth, the survivin antibodies used in the included studies do not discriminate between survivin isoforms, so the results may reflect increases in total survivin levels. We suggest that these preliminary findings warrant further analyses in the future.

Despite the above limitations, our meta-analysis presents evidence that survivin overexpression is associated with metastatic lymph node metastasis and poor prognosis in cervical carcinoma. Therefore, survivin may be a therapeutic target for cervical carcinoma. However, larger clinical studies must be performed to more thoroughly investigate the precise clinicopathological features associated with survivin.

## Supporting Information

S1 Checklist PRISMA Checklist. (DOC)

S1 Table Full Dataset. This table includes the data we used to conduct the analyses in this study.
(DOCX)

S2 Table Literature Quality Assessment. This study used the Newcastle-Ottawa Scale (NOS) for Assessing the Quality of Nonrandomized Studies in Meta-Analysis. Entries with a symbol represent earning one star, with the total number of stars in the right-most column.
(DOCX)

## References

[1] Colombo N, Carinelli S, Colombo A, Marini C, Rollo D, Sessa C. Cervical cancer: ESMO Clinical Practice Guidelines for diagnosis, treatment and follow-up. Ann Oncol. 2012;23: vii27–vii32. 10.1093/annonc/mds268 . 22997451

[2] Barbera L, Thomas G. Management of early and locally advanced cervical cancer. Semin Oncol. 2009;36: 155–169. 10.1053/j.seminoncol.2008.12.007 . 19332250

[3] Friedlander M, Grogan M, U.S. Preventative Services Task Force. Guidelines for the treatment of recurrent and metastatic cervical cancer. Oncologist. 2002;7: 342–347. . 12185296

[4] Jha K, Shukla M, Pandey M. Survivin expression and targeting in breast cancer. Surg Oncol. 2012;21: 125–131. 10.1016/j.suronc.2011.01.001 . 21334875

[5] Chen P, Zhu J, Liu DY, Li HY, Xu N, Hou M. Over-expression of survivin and VEGF in small-cell lung cancer may predict the poorer prognosis. Med Oncol. 2014;31: 775 10.1007/s12032-013-0775-5 . 24338338

[6] Lu CD, Altieri DC, Tanigawa N. Expression of a novel antiapoptosis gene, survivin, correlated with tumor cell apoptosis and p53 accumulation in gastric carcinomas. Cancer Res. 1998;58: 1808–1812. . 9581817

[7] Ikeguchi M, Ueda T, Sakatani T, Hirooka Y, Kaibara N. Expression of survivin messenger RNA correlates with poor prognosis in patients with hepatocellular carcinoma. Diagn Mol Pathol. 2002;11: 33–40. 10.1097/00019606-200203000-00007 . 11854600

[8] Ambrosini G, Adida C, Altieri DC. A novel anti-apoptosis gene, survivin, expressed in cancer and lymphoma. Nat Med. 1997;3: 917–921. 10.1038/nm0897-917 . 9256286

[9] Tamm I, Wang Y, Sausville E, Scudiero DA, Vigna N, Oltersdorf T, et al IAP-family protein survivin inhibits caspase activity and apoptosis induced by Fas (CD95), Bax, caspases, and anticancer drugs. Cancer Res. 1998;58: 5315–5320. . 9850056

[10] Li F, Ambrosini G, Chu EY, Plescia J, Tognin S, Marchisio PC, et al Control of apoptosis and mitotic spindle checkpoint by survivin. Nature. 1998;396: 580–584. 10.1038/25141 . 9859993

[11] Samuel T, Okada K, Hyer M, Welsh K, Zapata JM, Reed JC. cIAP1 localizes to the nuclear compartment and modulates the cell cycle. Cancer Res. 2005;65: 210–218. . 15665297

[12] Tran J, Master Z, Yu JL, Rak J, Dumont DJ, Kerbel RS. A role for survivin in chemoresistance of endothelial cells mediated by VEGF. Proc Natl Acad Sci U S A 2002;99: 4349–4354. 10.1073/pnas.072586399 . 11917134 PMC123651

[13] Stang A. Critical evaluation of the Newcastle-Ottawa scale for the assessment of the quality of nonrandomized studies in meta-analyses. Eur J Epidemiol. 2010;25: 603–605. 10.1007/s10654-010-9491-z . 20652370

[14] Parmar MK, Torri V, Stewart L. Extracting summary statistics to perform meta-analyses of the published literature for survival endpoints. Statist Med. 1998;17: 2815–2834. 10.1002/(SICI)1097-0258(19981230)17:24<2815::AID-SIM110>3.0.CO;2-8 . 9921604

[15] DerSimonian R, Laird N. Meta-analysis in clinical trials. Control Clin Trials. 1986;7: 177–188. 10.1016/0197-2456(86)90046-2 . 3802833

[16] Egger M, Davey Smith G, Schneider M, Minder C. Bias in meta-analysis detected by a simple, graphical test. BMJ. 1997;315: 629–634. 10.1136/bmj.315.7109.629 . 9310563 PMC2127453

[17] Lee JP, Chang KH, Han JH, Ryu HS. Survivin, a novel anti-apoptosis inhibitor, expression in uterine cervical cancer and relationship with prognostic factors. Int J Gynecol Cancer. 2005;15: 113–119. 10.1111/j.1048-891X.2005.15011.x . 15670305

[18] Zhu H, Hu Y, Shuai CX, Shi ZZ, Zhang HX. Expression of survivin, bcl-2 in cervical carcinoma and its association with HPV16/18 infection. Zhonghua Yi Xue Za Zhi. 2010;90: 469–473. . 20368071

[19] Lu H, Gan M, Zhang G, Zhou T, Yan M, Wang S. Expression of survivin, caspase-3 and p53 in cervical cancer assessed by tissue microarray: correlation with clinicopathology and prognosis. Eur J Gynaecol Oncol. 2010;31: 662–666. . 21319512

[20] Cao XQ, Lu HS, Zhang L, Chen LL, Gan MF. MEKK3 and survivin expression in cervical cancer: association with clinicopathological factors and prognosis. Asian Pac J Cancer Prev. 2014;15: 5271–5276. 10.7314/APJCP.2014.15.13.5271 . 25040987

[21] Liu HQ, Wang YH, Wang LL, Hao M. P16INK4A and survivin: diagnostic and prognostic markers in cervical intraepithelial neoplasia and cervical squamous cell carcinoma. Exp Mol Pathol. 2015;99: 44–49. 10.1016/j.yexmp.2015.04.004 . 25910412

[22] Lu S, Zhang B, Wang Z. Expression of survivin, cyclinD1, p21(WAF1), caspase-3 in cervical cancer and its relation with prognosis. J Huazhong Univ Sci Technolog Med Sci. 2005;25: 78–81. . 15934315 10.1007/BF02831393

[23] Wang M, Wang B, Wang X. [A novel antiapoptosis gene, survivin, bcl-2, p53 expression in cervical carcinomas]. Zhonghua Fu Chan Ke Za Zhi. 2001;36: 546–548. . 11769670

[24] Yaqin M, Runhua L, Fuxi Z. Analyses of Bcl-2, Survivin, and CD44v6 expressions and human papillomavirus infection in cervical carcinomas. Scand J Infect Dis. 2007;39: 441–448. 10.1080/00365540601105772 . 17464868

[25] Lu D, Qian J, Yin X, Xiao Q, Wang C, Zeng Y. Expression of PTEN and survivin in cervical cancer: promising biological markers for early diagnosis and prognostic evaluation. Br J Biomed Sci. 2012;69: 143–146. . 23310986

[26] Lang Y, Xiong YY, Chen HZ. [Association between expression of survivin and high-risk human papillomavirus infection in cervical cancer and precancerous tissues] Di Yi Jun Yi Da Xue Xue Bao. 2005;25: 1276–1279. . 16234108

[27] Wu SF, Zhang JW, Qian WY, Yang YB, Liu Y, Dong Y, et al Altered expression of survivin, Fas and FasL contributed to cervical cancer development and metastasis. Eur Rev Med Pharmacol Sci. 2012;16: 2044–2050. . 23280017

[28] Zhang LQ, Wang J, Jiang F, Xu L, Liu FY, Yin R. Prognostic value of survivin in patients with non-small cell lung carcinoma: a systematic review with meta-analysis. PLOS ONE. 2012;7: e34100 10.1371/journal.pone.0034100 . 22457815 PMC3311582

[29] Okada E, Murai Y, Matsui K, Isizawa S, Cheng C, Masuda M, et al Survivin expression in tumor cell nuclei is predictive of a favorable prognosis in gastric cancer patients. Cancer Lett. 2001;163: 109–116. 10.1016/S0304-3835(00)00677-7 . 11163114

[30] Li C, Li Z, Zhu M, Zhao T, Chen L, Ji W, et al Clinicopathological and prognostic significance of survivin over-expression in patients with esophageal squamous cell carcinoma: a meta-analysis. PLOS ONE. 2012;7: e44764 10.1371/journal.pone.0044764 . 23028610 PMC3459962

[31] Altieri DC, Marchisio PC. Survivin apoptosis: an interloper between cell death and cell proliferation in cancer. Lab Invest; J Tech Methods Pathol. 1999;79: 1327–1333. . 10576203

[32] Kim HS, Shiraki K, Park SH. Expression of survivin in CIN and invasive squamous cell carcinoma of uterine cervix. Anticancer Res. 2002;22: 805–808. . 12014654

[33] McKenzie JA, Liu T, Goodson AG, Grossman D. Survivin enhances motility of melanoma cells by supporting Akt activation and {alpha}5 integrin upregulation. Cancer Res. 2010;70: 7927–7937. 10.1158/0008-5472.CAN-10-0194 . 20807805 PMC2955769

[34] Kogo R, How C, Chaudary N, Bruce J, Shi W, Hill RP, et al The microRNA-218~Survivin axis regulates migration, invasion, and lymph node metastasis in cervical cancer. Oncotarget. 2015;6: 1090–1100. 10.18632/oncotarget.2836 . 25473903 PMC4359219

[35] Mahotka C, Wenzel M, Springer E, Gabbert HE, Gerharz CD. Survivin-deltaEx3 and survivin-2B: two novel splice variants of the apoptosis inhibitor survivin with different antiapoptotic properties. Cancer Res. 1999;59: 6097–6102. . 10626797

[36] Vandghanooni S, Eskandani M, Montazeri V, Halimi M, Babaei E, Feizi MA. Survivin-deltaEx3: a novel biomarker for diagnosis of papillary thyroid carcinoma. J Cancer Res Ther. 2011;7: 325–330. 10.4103/0973-1482.87038 . 22044815

[37] Span PN, Tjan-Heijnen VC, Heuvel JJ, de Kok JB, Foekens JA, Sweep FC. Do the survivin (BIRC5) splice variants modulate or add to the prognostic value of total survivin in breast cancer? Clin Chem. 2006;52: 1693–1700. 10.1373/clinchem.2006.071613 . 16873289

[38] Krieg A, Mahotka C, Krieg T, Grabsch H, Müller W, Takeno S, et al Expression of different survivin variants in gastric carcinomas: first clues to a role of survivin-2B in tumour progression. Br J Cancer. 2002;86: 737–743. 10.1038/sj.bjc.6600153 . 11875736 PMC2375298

[39] Meng H, Lu CD, Sun YL, Dai DJ, Lee SW, Tanigawa N. Expression level of wild-type survivin in gastric cancer is an independent predictor of survival. World J Gastroenterol. 2004;10: 3245–3250. 10.3748/wjg.v10.i22.3245 . 15484293 PMC4572288

[40] Yamada Y, Kuroiwa T, Nakagawa T, Kajimoto Y, Dohi T, Azuma H, et al Transcriptional expression of survivin and its splice variants in brain tumors in humans. J Neurosurg. 2003;99: 738–745. 10.3171/jns.2003.99.4.0738 . 14567610

[41] Werner TA, Tamkan-Ölcek Y, Dizdar L, Riemer JC, Wolf A, Cupisti K, et al Survivin and XIAP: two valuable biomarkers in medullary thyroid carcinoma. Br J Cancer. 2016;114: 427–434. 10.1038/bjc.2016.5 . 26882066 PMC4815780

[42] Kawasaki H, Altieri DC, Lu CD, Toyoda M, Tenjo T, Tanigawa N. Inhibition of apoptosis by survivin predicts shorter survival rates in colorectal cancer. Cancer Res. 1998;58: 5071–5074. . 9823313

[43] Hong JH, Tsai CS, Wang CC, Lai CH, Chen WC, Lee SP, et al Comparison of clinical behaviors and responses to radiation between squamous cell carcinomas and adenocarcinomas/adenosquamous carcinomas of the cervix. Chang Gung Med J. 2000;23: 396–404. . 10974754

[44] Engels K, Knauer SK, Metzler D, Simf C, Struschka O, Bier C, et al Dynamic intracellular survivin in oral squamous cell carcinoma: underlying molecular mechanism and potential as an early prognostic marker. J Pathol. 2007;211: 532–540. 10.1002/path.2134 . 17334981

[45] Suzuki Y, Oka K, Yoshida D, Shirai K, Ohno T, Kato S, et al Correlation between survivin expression and locoregional control in cervical squamous cell carcinomas treated with radiation therapy. Gynecol Oncol. 2007;104: 642–646. 10.1016/j.ygyno.2006.10.005 . 17141299

[46] Jacquemier J, Molès JP, Penault-Llorca F, Adélaide J, Torrente M, Viens P, et al p53 immunohistochemical analysis in breast cancer with four monoclonal antibodies: comparison of staining and PCR-SSCP results. Br J Cancer. 1994;69: 846–852. 10.1038/bjc.1994.164 . 7514027 PMC1968919

