# Supplementary material for: Survivin Overexpression Is Associated with Aggressive Clinicopathological Features in Cervical Carcinoma: A Meta-Analysis
Source: PLoS One. 2016 Oct 20;11(10):e0165117. doi: 10.1371/journal.pone.0165117 (PMC5072693; doi:10.1371/journal.pone.0165117)
Supplement: S1 Table — This table includes the data we used to conduct the analyses in this study. (DOCX) [file pone.0165117.s002.docx]

| **First Author** | | Lee J.P | H.Zhu | H.lu | X.Q.Cao | H.Q.Liu | S.Lu | M.Wang | Y.Q.Mu | D.Lu | Y.Lan | S.F.WU |
| --- | --- | --- | --- | --- | --- | --- | --- | --- | --- | --- | --- | --- |
| **Year** | | 2005 | 2010 | 2010 | 2014 | 2015 | 2005 | 2001 | 2007 | 2012 | 2005 | 2012 |
| **Study Design** | | Retrospective cohort | Retrospective cohort | Retrospective cohort | Retrospective cohort | Retrospective cohort | Retrospective cohort | Retrospective cohort | Retrospective cohort | Retrospective cohort | Retrospective cohort | Retrospective cohort |
| **Country** | | Korea | China | China | China | China | China | China | China | China | China | China |
| **Total cases** | | 64 | 101 | 142 | 116 | 80 | 51 | 69 | 75 | 59 | 41 | 67 |
| **Total controls** | | 11 | 20 | 35 | 35 | 30 | 10 | 10 | 25 | 10 | 10 | 20 |
| **No.normal** | | 7/11 | 0/20 | 0/35 | 0/35 | 2/30 | 3/10 | 0/10 | 1/25 | 1/10 | 0/10 | 0/20 |
| **No.cancer** | | 51/53 | 63/81 | 90/107 | 72/81 | 40/50 | 32/41 | 41/59 | 45/50 | 35/49 | 26/31 | 32/47 |
| **Method** | | IHC | RT-PCR | IHC | RT-PCR | IHC | IHC | IHC | IHC | IHC | IHC | IHC |
| **Sample sources** | | SCC&ADC | SCC&ADC | SCC&ADC | SCC&ADC | SCC&ADC | SCC&ADC | SCC | SCC&ADC | SCC | SCC&ADC | SCC |
| **Tumor stage** | | Ⅰ-Ⅳ | Ⅰ-Ⅲ | Ⅰ-Ⅳ | Ⅰ-Ⅳ | Ⅰ-Ⅱ | Ⅰ-Ⅳ | Ⅰ-Ⅲ | Ⅰ-Ⅳ | Ⅰ-Ⅳ | Ⅰ-Ⅳ | Ⅰ-Ⅲ |
| **Cut off** | | 5% | 5% | 5% | 10% | 5% | 5% | 10% | 5% | 10% | 10% | 10% |
| **HR estimate** | | Sur.curve | Sur.curve | HR |  | HR |  |  |  |  |  |  |
| **Location** | | Nucleus/Cyto | Cyt. | Cyt. | Cyt. | Nu.&Cyt. | Cyt. | Cyt. | Cyt. | Cyt. | Nu.&Cyt. | Cyt. |
| **Lymph node metastasis** | **negtative** | 9/53 | 15/33 | 64/81 | 66/83 | 16/24 | 24/32 | N/A | 15/24 | 15/28 | N/A | 7/17 |
|  | **postive** | 44/53 | 19/21 | 26/26 | 24/24 | 24/26 | 8/9 | N/A | 25/26 | 20/21 | N/A | 25/30 |
| **FIGO stages** | **IIB** |  | N/A | 65/81 | 65/81 | 26/36 | 23/30 | 4/8 | 25/27 | 11/20 | 21/26 | 29/44 |
|  | **III-IVA** |  |  | 25/25 | 25/26 | 14/14 | 9/11 | 36/44 | 15/23 | 24/29 | 4/5 | 3/3 |
| **tumour grade** | **high** |  | 42/59 | 59/65 | 57/65 | 29/39 | 19/22 | 18/31 | 22/23 | 22/35 | 18/21 | 30/44 |
|  | **low** |  | 21/22 | 13/16 | 15/16 | 11/11 | 13/19 | 23/28 | 18/27 | 13/14 | 8/8 | 2/3 |
| **Size of tumor** | **<4** |  |  |  |  | 30/37 |  |  |  | 18/30 |  |  |
|  | **>4** |  |  |  |  | 10/13 |  |  |  | 17/19 |  |  |
| **Stromal involvment** | **negtative** |  |  | 19/28 | 19/28 | 19/23 |  |  |  |  |  |  |
|  | **postive** |  |  | 71/79 | 71/79 | 21/27 |  |  |  |  |  |  |
