# Supplementary material for: Survivin Overexpression Is Associated with Aggressive Clinicopathological Features in Cervical Carcinoma: A Meta-Analysis
Source: PLoS One. 2016 Oct 20;11(10):e0165117. doi: 10.1371/journal.pone.0165117 (PMC5072693; doi:10.1371/journal.pone.0165117)
Supplement: S2 Table — This study used the Newcastle-Ottawa Scale (NOS) for Assessing the Quality of Nonrandomized Studies in Meta-Analysis. Entries with a symbol represent earning one star, with the total number of stars in the right-most column. (DOCX) [file pone.0165117.s003.docx]

| **First Author** | Lee J.P | H.Zhu | H.lu | X.Q.Cao | H.Q.Liu | S.Lu | M.Wang | Y.Q.Mu | D.Lu | Y.Lan | S.F.WU |
| --- | --- | --- | --- | --- | --- | --- | --- | --- | --- | --- | --- |
| **Year** | 2005 | 2010 | 2010 | 2014 | 2015 | 2005 | 2001 | 2007 | 2012 | 2005 | 2012 |
| **Study Design** | Retrospective cohort | Retrospective cohort | Retrospective cohort | Retrospective cohort | Retrospective cohort | Retrospective cohort | Retrospective cohort | Retrospective cohort | Retrospective cohort | Retrospective cohort | Retrospective cohort |
| **Defining cases (i.e. survivin expression )** | independently measured* | independently measured* | independently measured* | independently measured* | independently measured* | independently measured* | independently measured* | independently measured* | independently measured* | independently measured* | independently measured* |
| **Cases appropriately selected** | well-selected* | selected from same source as controls* | well-selected* | well-selected* | well-selected* | well-selected* | Well-selected* | Well-selected* | well-selected* | well-selected* | well-selected* |
| **Source of controls** | Hospital controls | Hospital controls | Hospital controls | Hospital controls | Hospital controls | Hospital controls | Hospital controls | Hospital controls | Hospital controls | Hospital controls | Hospital controls |
| **Defining controls** | Yes* | Yes* | Yes* | Yes* | Yes* | Yes* | Yes* | Yes* | Yes* | Yes* | Yes* |
| **Controlled for tissues sources** | Paraffinem-bedded  blocks * | Paraffinem-bedded  blocks * | Paraffinem-bedded  blocks * | Paraffinem-bedded  blocks and cervical tissues* | Paraffinem-bedded  blocks * | Paraffinem-bedded  blocks * | Paraffinem-bedded  blocks * | formalin-fixed and paraffinembedded  tissue blocks * | Paraffinem-bedded  blocks * | Paraffinem-bedded  blocks * | Paraffinem-bedded  blocks * |
| **Controlled for pathological features** | yes* | In part | yes* | yes* | yes* | In part | In part | In part | Yes* | In part | yes* |
| **Assessment of outcome/exposure** | Secure record* | Secure record* | Secure record* | Secure record* | Secure record* | Secure record* | record link* | record link* | Secure record* | Secure record* | Secure record* |
| **Method for ascertaining cases/controls the same** | Yes* | Yes* | Yes* | Yes* | Yes* | Yes* | Yes* | yes* | Yes* | Yes* | Yes* |
| **Non-response rate within 10%** | Yes* | Yes* | Yes* | Yes* | Yes* | Does not mention | Does not mention | Does not mention | Does not mention | Does not mention | Does not mention |
| **Total Stars** | 8 | 7 | 8 | 8 | 8 | 6 | 6 | 6 | 7 | 6 | 7 |
